# Nuclear Receptor Signaling Atlas: Opening Access to the Biology of Nuclear Receptor Signaling Pathways

**DOI:** 10.1371/journal.pone.0135615

**Published:** 2015-09-01

**Authors:** Lauren B. Becnel, Yolanda F. Darlington, Scott A. Ochsner, Jeremy R. Easton-Marks, Christopher M. Watkins, Apollo McOwiti, Wasula H. Kankanamge, Michael W. Wise, Michael DeHart, Ronald N. Margolis, Neil J. McKenna

**Affiliations:** 1 Dan L. Duncan Comprehensive Cancer Center Biomedical Informatics Group, One Baylor Plaza, Houston, Texas, United States of America; 2 Department of Molecular and Cellular Biology, Baylor College of Medicine, One Baylor Plaza, Houston, Texas, United States of America; 3 National Institute of Diabetes, Digestive and Kidney Diseases, Division of Diabetes and Metabolic Diseases, Bethesda, Maryland, United States of America; 4 Nuclear Receptor Signaling Atlas (NURSA) Informatics Hub; Univeristy of California Riverside, UNITED STATES

## Abstract

Signaling pathways involving nuclear receptors (NRs), their ligands and coregulators, regulate tissue-specific transcriptomes in diverse processes, including development, metabolism, reproduction, the immune response and neuronal function, as well as in their associated pathologies. The Nuclear Receptor Signaling Atlas (NURSA) is a Consortium focused around a Hub website (www.nursa.org) that annotates and integrates diverse ‘omics datasets originating from the published literature and NURSA-funded Data Source Projects (NDSPs). These datasets are then exposed to the scientific community on an Open Access basis through user-friendly data browsing and search interfaces. Here, we describe the redesign of the Hub, version 3.0, to deploy “Web 2.0” technologies and add richer, more diverse content. The Molecule Pages, which aggregate information relevant to NR signaling pathways from myriad external databases, have been enhanced to include resources for basic scientists, such as post-translational modification sites and targeting miRNAs, and for clinicians, such as clinical trials. A portal to NURSA’s Open Access, PubMed-indexed journal *Nuclear Receptor Signaling* has been added to facilitate manuscript submissions. Datasets and information on reagents generated by NDSPs are available, as is information concerning periodic new NDSP funding solicitations. Finally, the new website integrates the Transcriptomine analysis tool, which allows for mining of millions of richly annotated public transcriptomic data points in the field, providing an environment for dataset re-use and citation, bench data validation and hypothesis generation. We anticipate that this new release of the NURSA database will have tangible, long term benefits for both basic and clinical research in this field.

## Introduction

Nuclear receptors (NRs) represent the largest family of metazoan transcription factors and function as ligand-dependent sensors for a diverse set of fat-soluble hormones, vitamins, and dietary lipids [1]. NRs and their ligands mediate the expression of genes involved in a broad range of reproductive, developmental, metabolic and immune response programs. As a corollary, members of the NR superfamily encompass one of the most successful targets for drugs available to treat a multitude of therapeutic indices, including cancer, diabetes, obesity, hypertension, cardiovascular disease, senescent illnesses and the metabolic syndrome [2]. In addition, the impact upon NR signaling pathways of xenobiotics [3] and endocrine disrupting chemicals [4] is gaining increased attention. Coregulators constitute a group of currently ~400 published molecules that have been shown to be required by one or more NRs for efficient activation or repression of gene expression [5, 6]. In many cases these proteins had been previously characterized in other contexts and a substantial body of data already exists to implicate them in a variety of disease states and animal model aberrations. Coregulators are known to participate in many of the composite steps required for regulation of gene expression from transcription to post-translational modification [7]. Like NRs, coregulators have been implicated in myriad disease states and offer an additional leverage point for the development of therapeutics [8].

Attesting to the broad importance of NR signaling in human physiology and disease, the Nuclear Receptor Signaling Atlas (NURSA) Consortium has been supported by seven different NIH institutes since its inception in 2002 [9], with the majority of funding originating from the NIDDK. The mission of NURSA is to accrue, develop, and communicate information that advances our understanding of the roles of NRs and their coregulators in processes, diseases and conditions in which they play an integral role. It accomplishes these goals through three mechanisms. **(1) A Hub website and data analysis portal**. The NURSA Hub website, www.nursa.org, aggregates data on molecules, pathways, drugs, diseases and phenotypes associated with NRs and coregulators from dozens of public databases, serving as a “one-stop shop” for the NR community. In addition, it disseminates NDSP datasets and reagent information on an Open Access basis. The Hub also contains data analysis tools, such as Transcriptomine, which aggregates and annotates NR-relevant transcriptomic datasets from the published literature and exposes these to the research community. **(2) *Nuclear Receptor Signaling*** NURSA’s Open Access electronic journal, *Nuclear Receptor Signaling* (*NRS*), affords the scientific community the opportunity to contribute primary research, perspectives, reviews and methods to the NURSA knowledgebase, and exposes the NURSA resource to new visitors through PubMed searches. **(3) NURSA Data Source Projects** Each year, NURSA solicits proposals for 1–2 year NURSA Data Source Projects (NDSPs) that provide input of novel, important data through the generation of ‘omics scale datasets in topic areas selected after consultation with the scientific community. These diverse but complementary components are designed to afford the community a more complete understanding of the role of NRs in human physiology and in the pathophysiology myriad disease conditions. Although other web-based NR-centric resources exist, including NuclearDB [10] and the Nuclear Receptor Resource [11], none combines NURSA’s commitment to novel ‘omics research funding; dataset curation annotation and mining; and a variety of mechanisms to establish two-way communicate with the broader research community. Here, we describe the current status of the Consortium and the NURSA 3.0 Hub website available at www.nursa.org.

## Materials and Methods

### Application and Databases

NURSA (current version 3.0.2) is a Java Enterprise Edition 6, web-based application built around an Oracle 11g database for structured data and a MongoDB store for datasets from NDSPs that can be downloaded by the public, images and other miscellaneous files. The application runs in JBoss 7 on Red Hat Enterprise Linux, though other Unix-based operating systems (e.g, CentOS) may be utilized. For additional security, Apache middleware acts as a proxy to filter incoming requests to JBoss only on specified ports. The system uses the model-view-controller architecture to manage its various components. The NURSA model uses Hibernate implementation of the JPA (Java Persistence API) for Entity persistence in its Oracle 11g database. The views in the UI were created using a FlatUI toolkit with a combination of JavaScript, D3.JS, AJAX, HTML5 and CSS3. Java Server Faces (JSF), and Primefaces are the primary technologies behind the UI. The website is device-aware, and its UI will automatically reorient its components on mobile devices and ultraportable laptops to optimize user experience. NURSA has been optimized for Firefox 24+, Chrome 30+, Safari 5.1.9+ and Internet Explorer 9+ with validations performed in BrowserStack and load testing in LoadUIWeb.

### Data Extraction and Aggregation from Public Databases

Thousands of publicly-available databases exist that contain information relevant to NR-and coregulator genes, their encoded mRNAs and proteins, and other pertinent clinical and biomedical data. It is not feasible to incorporate data from all, nor desirable as many resources may not have appropriately stringent quality control and assurance mechanisms in place. To aggregate public data for the NURSA 3.0 Molecule and Clinical/Drug pages, we prioritized those resources already present in the NURSA 2.0 website (e.g, NCBI Gene, UniProt, Phosphosite) and supplemented these with well-established public resources that have established data standards and quality control procedures (e.g. DrugBank, ClinicalTrials.gov). Prioritization of aggregation from these resources was supported by input from our Beta Testing Group, an international, external panel of bench scientist volunteers. For each core resource, methods to obtain data (e.g, application programming interfaces [APIs]), database dumps, flat files) are reviewed using published documentation from the resource and the metadata mapped to the NURSA object model. Data are then retrieved, analyzed and committed to the NURSA database via custom ETL (extract, transform, load) processes that extract relevant data and annotations, transform them to the NURSA 3.0 model, and load them into the backend database tables. Depending on the source data type and structure, ETL mechanisms are selected based upon the available data structure/data type, including shell scripts for simple text manipulation of flat files, database-mediated processes using tools within Oracle Enterprise Manager, or Java code. ETL scripts are run first on a subset of data, and the results validated within the database and through the user interface by the team. Test scripts, including user interface-based (UI) skits via BrowserStack, are utilized for iterative testing. Once validated, ETL processes are run on the entire dataset, and test script validation performed. The semi-automated data extraction and analysis pipeline for Transcriptomine that is coupled with expert human curation and annotation has been described elsewhere [12]. Briefly an automated script searches MEDLINE for publications containing transcriptomic datasets of relevance to the NR community, which are placed in an expert human curator’s queue. The curator reviews the publication for suitability, then obtains datasets from public repositories, where available, or from supplemental material in journal articles. Data are processed through a standard pipeline then annotated using standard ontologies such as NCBI Taxon (species) and small molecule (PubChem) taxonomies, Drugbank and an RNA Source ontology. These annotated, processed data are then exposed through the Transcriptomine user interface for mining by end users.

### Website Development: Process and Implementation

The Hub development team uses a modified Agile development approach, in which a mid- and long-term development accomplishments are mapped out NURSA, prioritized into 1–2 month goals, and each goal broken down into specific tasks that are grouped into 2–3 week development activities, referred to as sprints. After each sprint, code is tested internally, then released to the beta site (beta.nursa.org). The code is demonstrated via webinar to the Beta Testing Group, which is given 1–2 weeks to review and provide feedback or ask for additional enhancements, features or bug fixes. This feedback is prioritized by the NURSA team, and any immediate needs addressed prior to moving the code to the production NURSA Hub website, www.nursa.org.

### UML Models, Code and Documentation

The NURSA logical model was created using Enterprise Architect. End user documentation, README files for deployment and a deployment guide were created in Microsoft Word or similar word processing programs. All documentation is available in the NURSA Github environment [13]. Full database dumps and a snapshot of the NURSA virtual machine environment are available upon request.

## Results

A 2012 survey sent to the NURSA listserv, which is composed of approximately 4,000 global Hub end users, showed that while the rich content was appreciated, substantial modifications were warranted to improve usability, mobile device compatibility and inclusion of clinical and translational data. To maximize utility and value to the community, demonstrate responsiveness to end user feedback, and modernize the underlying technology of the website, we embarked upon the development of NURSA 3.0, which entirely replaces the previous NURSA informatics infrastructure. The NURSA 3.0 Hub has maintained and expanded its popular NURSA Molecule Pages, incorporated the biologist-friendly transcriptomics data mining tool Transcriptomine, and redesigned the peer-reviewed, MEDLINE and PubMed Central-indexed journal *Nuclear Receptor Signaling* (NRS). The look and feel of the site has been modernized to facilitate site navigation and data discovery, and to enhance accessibility by users on mobile devices. To populate these webpages, NURSA 3.0 aggregates and integrates information from dozens of disparate biomedical databases–an activity that would take hours to days for many researchers–to provide an instant, holistic overview of NRs, ligands, coregulators, tissue-specific target genes other biological entities. In addition, to minimize future maintenance costs, we have developed a streamlined semi-automated data processing and loading pipeline that maintains the currency and relevance of NURSA 3.0 content.

### NURSA 3.0 Model

At the core of the NURSA 3.0 object model, as with previous iterations of the web resource, is the central dogma of molecular biology, namely, genes, which are linked to their mRNA transcripts, which are in turn mapped to the proteins they encode. Layered on top of this information flow are more complex biological entities such as post-translational modifications, targeting miRNAs, interactions, pathways, diseases and drugs. The model is highly abstracted to centralize concepts such as molecular annotations for different classes of molecules (e.g., genes, mRNAs, proteins), as well as to synonyms for these concepts. URLs link to external resources that are managed within a small number of classes containing standard attributes to facilitate performance in the face of a large and expanding data pool (see ref. 13 for deposited code and associated material). For example, although NURSA’s primary gene identifiers (IDs) are Entrez IDs, other gene identifiers, gene names and synonyms are stored within a Synonym object. Data on over 20 common model organism species (human, mouse, rat, fruit fly, zebrafish, dog, cow, rabbit, chicken, chimpanzee, Rhesus monkey, African malaria mosquito, Thale cress, rice, fission yeast, baker’s yeast, *N*. *crassa*, *C*. *elegans*, *K*. *lactis*, *E*. *gossypii*, and *M*. *oryzae*) whose genomes have been sequenced and are available from NCBI have been extracted and loaded into the NURSA database as a supporting foundation for data from dozens of other resources such as miRbase, UniProtKB, Phosphosite, DrugBank, ClinicalTrials.gov, and others. NRs and coregulators are specifically flagged within the model to denote which of the tens of thousands of genes, and by extension other molecule types, map to canonical NRs and coregulators across different species. Though these flags allow for targeted exposure of data within the core model that are focused on NR-relevant content, the model was purposely built to be readily adapted to other purposes by groups who may be interested in reusing the open source software under an LGPL license with appropriate attribution. By changing the flags to tag other sequence-defined superfamilies or functional molecular groups of interest (e.g. cytochrome P450 enzymes, integrins, kinases) and updating the user interface, those molecules, as well as the aggregated basic and clinical data within the underlying core model, will be exposed. Tagging with universal identifiers significantly enhances the ability to find and re-use the data and is consistent with emerging Big Data to Knowledge (BD2K) efforts underway at the National Institutes of Health [14].

### Hub Website Organization

#### Home Page

**Fig 1:**
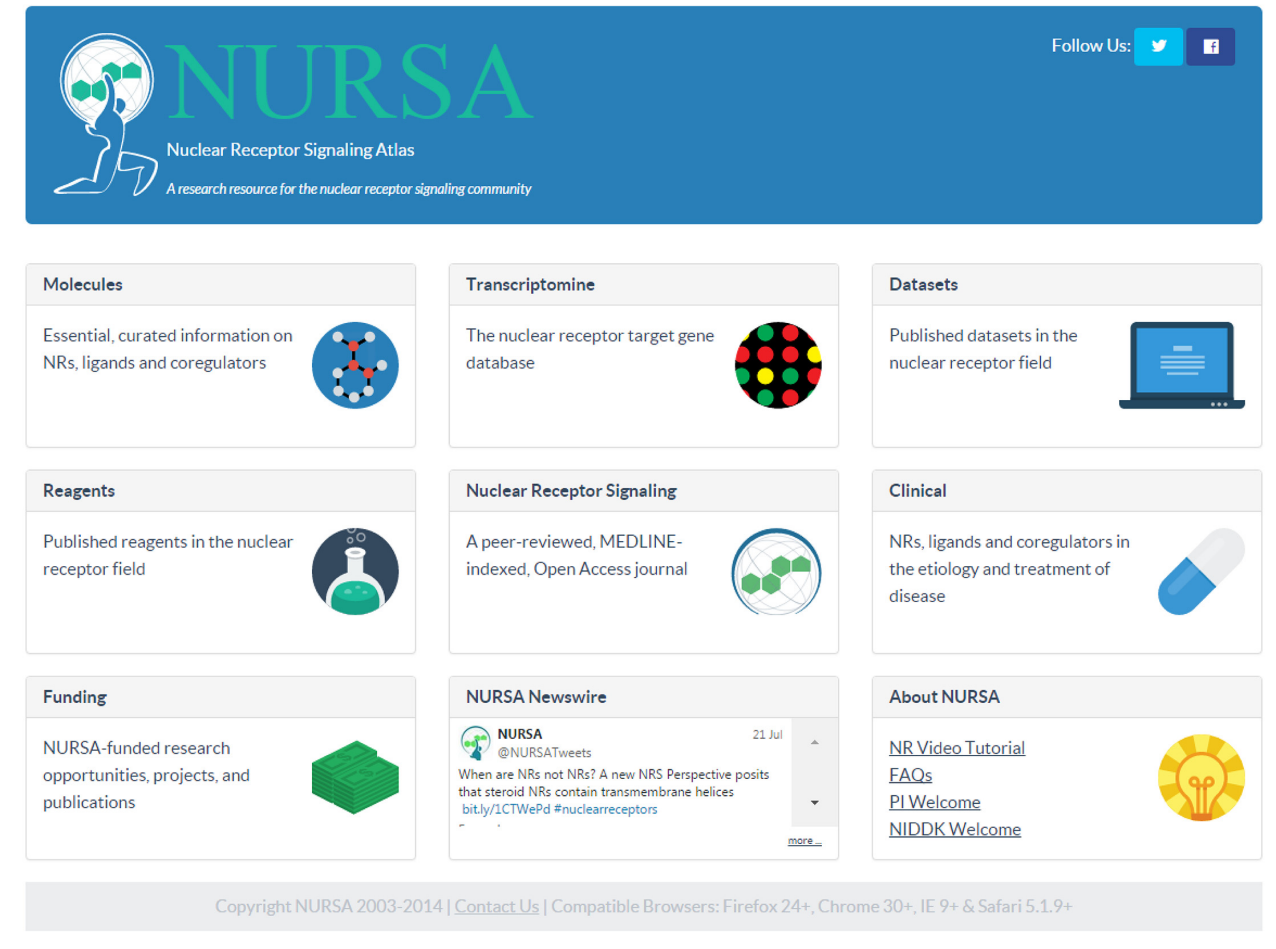
The NURSA 3.0 Homepage. All data within NURSA are now readily accessible through nine sections within the homepage. Webpages within each NURSA section contain links to pertinent related data within NURSA 3.0, e.g., a Molecule Page will contain links to clinical trials within the Clinical section, and links to external online resources to allow end users to rapidly discover and explore information. Reprinted under a CC BY license, with permission from the Nuclear Receptor Signaling Atlas, original copyright 2015.

The NURSA 3.0 home page (Fig 1) is divided into nine interlinked sections, each of which has its own detail box on the homepage: Molecules, Transcriptomine and other Tools, Datasets, Reagents, *Nuclear Receptor Signaling*, Clinical, Funding, Newswire and About. By clicking on any of these boxes, end users will navigate directly to the landing page for each functional section in order to browse, search or mine the data therein. The footer provides contact information for end users to submit questions and other support requests, as well as a listing of current compatible web browsers and versions.

#### All Molecules Page

**Fig 2:**
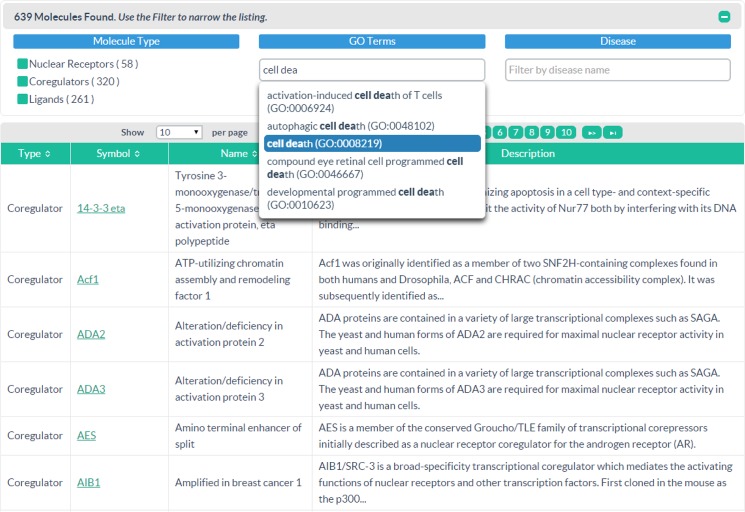
The All Molecules Page. On the All Molecules Page, end users can either browse nuclear receptors, coregulators and ligands, or narrow down the list of molecules using an Amazon-like filter module. In this example, the user enters the GO term “cell death” in the filter module to retrieve molecules mapping to that term. Reprinted under a CC BY license, with permission from the Nuclear Receptor Signaling Atlas, original copyright 2015.

The All Molecules Page (Fig 2) displays the molecule Type (e.g, NR, coregulator or ligand), Gene Symbol or Ligand Synonym, Name, and Description. Molecule Pages for any record can be reached by clicking on a Gene/Ligand Symbol hyperlink in the All Molecules Page. End users can browse through the table via sort buttons within column headings or navigation buttons at the bottom. Alternatively, an Amazon-like interface filters the list of molecules by specifying type (Nuclear receptor, coregulator, ligand) or by typing GO Terms or Diseases in autosuggest text boxes. To jump directly to a specific page, the user can type a molecule name, symbol, or synonym, then select from a list of suggested terms to go directly to a Molecule Page. A significant advance on previous functionality is a filter module that allows users to find molecules mapping to Gene Ontology (GO) categories or disease terms (e.g. Online Mendelian Inheritance in Man, OMIM) (Fig 2). In this way, users who are not familiar with NRs or coregulators, but are rather seeking to identify those that are relevant to their specific biological process or disease of interest, have a familiar entrée into the NURSA knowledge mine.

#### Molecule Pages

**Fig 3:**
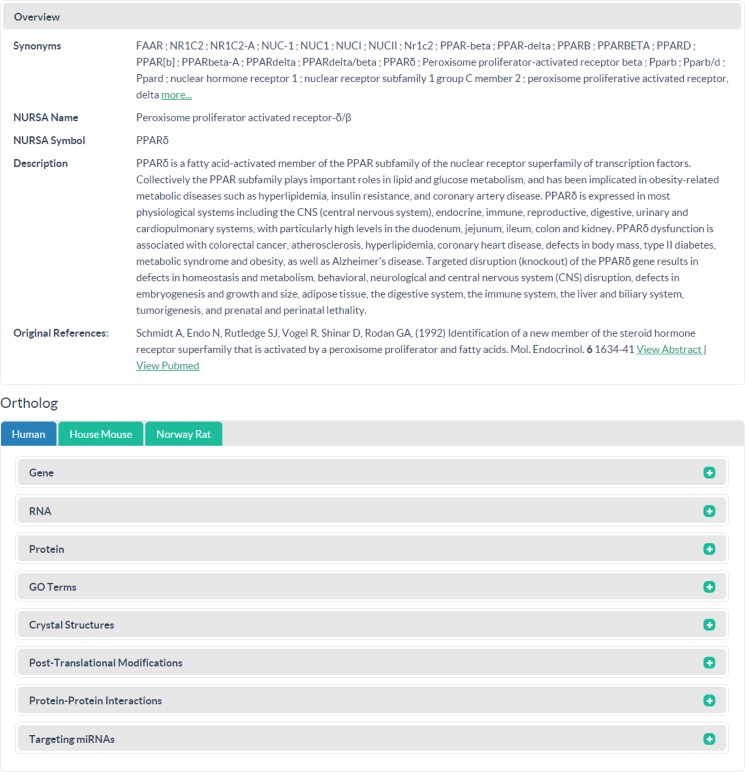
The Molecule Page. The Molecule Pages aggregate data from numerous external basic and clinical information resources, as well as public and NURSA-funded datasets, reagents and other entities. Reprinted under a CC BY license, with permission from the Nuclear Receptor Signaling Atlas, original copyright 2015.

Molecule Pages can be directly reached through the All Molecules search function and through hyperlinks on other NURSA 3.0 sections, including Transcriptomine query results. An overview section is present at the top of every Molecule Page to provide a listing of common synonyms for the gene and its homologs, the preferred NURSA gene name and symbol, a NURSA-curated description of the gene and, for NRs and coregulators, a NURSA-curated listing of seminal publications describing the cloning and characterization of the specific gene product (Fig 3). Genes are commonly cloned and characterized under a number of different names or in different functional contexts, and searches for any of these synonyms will resolve to a single Molecule Page. In the event that an end user’s search term is a synonym and not an identical match to the preferred NURSA gene symbol, orange text will appear at the top of the page explaining the relationship. Underneath the overview section, orthologue specific information for genes, RNAs, proteins, crystal structures and other data types are indexed by a listing of species vertical navigation menu at left. End users can toggle among human, mouse, rat and, for some genes where matching Dataset records exist, fruit fly. Given that data on nearly 20 other species are present in NURSA, the database can scale to encompass additional taxonomies, for example, when a new species is represented in a NDSP-generated datasets or in a Transcriptomine-annotated gene list.

In the Molecule Page user interface, expandable species-specific subsections include Gene, RNA, Protein, GO Terms, Post-Translational Modifications, Crystal Structures, Protein-Protein Interactions and Targeting miRNAs. Each section contains aggregated data from public data repositories, including popular model organism databases, with links to pertinent webpages within those resources and/or NURSA NDSPs. For example, when human is selected from the species navigation listing, the Gene subsection contains the NCBI official gene symbol and links to Ensembl, HGNC (HUGO Gene Nomenclature Committee), HPRD (Human Protein Reference Database); NCBI Gene; NCBI Epigenomic Tracks; OMIM; and VEGA (Vertebrate Genome Annotation Database) Genome Browser Viewer. When house mouse is selected, the same section contains the species-specific NCBI official gene symbol plus a link to Ensembl, MGI (Mouse Genome Informatics database), NCBI Gene, NCBI Epigenomic Tracks, and VEGA Viewer. Data may be visualized on popular genome browsers and through the JSMol crystal structure viewer by clicking the View button within the Crystal Structures subsection. With the JSMol viewer, end users may choose a representation rendering (e.g, ribbon, space filling, ball and stick) and use their mouse to rotate the structure in three dimensions. Within the Post-Translational Modifications subsection, modified amino acid residues within a given protein amino acid sequence are highlighted in red lowercase text, with the known modification type at that numbered residue also given with a RefSeq identifier for cross-referencing purposes. Many miRNAs have been predicted to target various genes, but NURSA shows only miRNAs that have been experimentally validated to target a given gene product within its Targeting miRNAs subsection.

Beneath the ortholog-specific sections, additional data for Datasets, Reagents, Diseases, Clinical Trials, Animal Models, Drugs and Literature are available. Annotated public or NDSP-funded datasets are displayed in the Datasets section. Where no NDSP datasets have generated data pertinent to a specific gene, however, only links to our data mining tools will be present within this section. Similarly, information on animal models and reagents generated by NURSA-funded NDSPs or that are available from other sources is contained in the Animal Model and Reagents sections. Finally, the Diseases, Drugs and Clinical Trials sections contain pertinent data from DrugBank, ClinicalTrials.gov and OMIM.

#### Transcriptomine

The prevailing model of scientific communication, the journal article, excels for the promulgation of mechanistic or functional dissections of biological phenomena, but is a relatively inefficient vehicle for the distribution and dissemination of data points in discovery-driven ‘omics datasets. Such datasets typically exist as abridged, informatically-opaque gene lists in journal articles, as archived raw files in formats challenging to the average bench researcher, or are simply unavailable in any form. We have previously described the development of the Transcriptomine tool, which allows users to mine transcriptomic datasets of relevance to the NR field [12]. Transcriptomine integrates gene lists from NDSP datasets with a much larger data number of datasets from the published literature and public repositories. By aggregating these datasets in a central database, processing them in a pipeline and annotating the data points using modern community standards and ontologies, Transcriptomine provides a vast resource for data validation, hypothesis generation and model testing. Consonant with NURSA’s commitment to high standards of usability, Its simple query interface obviates the need for informatics expertise on the part of the user to mine the database. Further, it allows users to leverage data points that are present within published datasets, but were not highlighted within the text of the original paper with which they were associated. This feature allows for rapid *in silico* hypothesis generation through discovery of previously-published, yet still largely occult, data.

A typical example is provided by the oncogene *PIM1*, which encodes a member of the serine/threonine family of kinases, and which a 2013 paper described as a novel estrogen-regulated gene [15]. There were in fact ten different data points from ten distinct datasets, some as long ago as 2004, which contained data points reflecting regulation of PIN1 by 17β-estradiol (Table 1). Corroborating these data points, are additional datasets preceding the 2013 publication that reflect induction of PIM1 by 4-nonylphenol (4NP) and bisphenol A (BPA), which are known EDCs targeting 17βE2-regulated pathways. Although additional mechanistic characterization was carried out in the 2013 paper, the argument can be reasonably made that exposure of these data points in a searchable database like Transcriptomine at the time of their original publication would have accelerated the characterization of PIN1 as a target for the 17βE2 signaling pathway. Moreover, the search suggests the testable research hypothesis that the toxicity of 4NP and BPA may be due in part to their upregulation of the *PIM1* oncogene.

**Table 1:** Data points reflecting induction of the *PIM1* oncogene by 17βE2 and by endocrine-disrupting chemicals targeting estrogenic pathways. All data points predate the 2013 paper [15] describing PIM1 as a novel estrogen target gene. Accession ID: identifier in original parent archive; OE, overexpression; Veh, Vehicle.

Biosample	Experiment	Year	Accession ID	Ref
MCF-7	17βE2 v Veh	2008	Not deposited	[16]
MCF-7-AKT	17βE2 v Veh	2008	Not deposited	[16]
MCF-7	17βE2 v Veh	2011	Not deposited	[17]
Uterus	17βE2 v Veh	2004	GSE2195	[18]
Uterus	17βE2 v Veh	2004	GSE2195	[18]
Uterus	17βE2 v Veh	2004	GSE2195	[18]
Uterus	17βE2 v Veh	2010	GSE23072	[19]
Uterus	17βE2 v Veh	2010	GSE23072	[19]
Uterus	17βE2 v Veh	2010	GSE23072	[19]
Testis	17βE2 v Veh	2009	GSE17553	[20]
Vagina	17βE2 v Veh	2008	GSE11622	[21]
MDA-MB-231	ERα/ESR1 + 17βE2 v 17βE2	2009	GSE9757	[22]
MDA-MB-231	ERα/ESR1 + 17βE2 v ERα/ESR1	2005	GSE2251	[23]
MCF-7	ERβ/ESR2 OE + 17βE2 v ERβ/ESR2 OE + Veh	2006	GSE4006	[24]
Hepatocytes	4NP v Veh	2009	E-MEXP-2539	[25]
Hepatocytes	4NP v Veh	2009	E-MEXP-2539	[25]
Ishikawa	BPA v Veh	2010	GSE17264	[26]
Ishikawa	BPA v Veh	2010	GSE17264	[26]

There are currently of the order of 25 million total data points from 360 datasets, representing at least ±2-fold difference in expression with a P-value of ≤0.05, that are available to query in Transcriptomine. End users may query by first specifying their gene or genes of interest through entering a single approved gene symbol or any NCBI-recognized synonym, OMIM disease term, GO term or by uploading a list of gene symbols. Results may be filtered further by specifying relative fold-change values, statistical measurements, species, biosample (tissue or cell line), and perturbing molecule. End users may also select a length of time that the tissues/cells were exposed to a given perturbing molecule. All query results are available for download within Excel up to the limit of the program (~660,000 results). Larger queries or copies of the entire database are available upon request through NURSA support. Q ueries that return under 500 records can be downloaded in Excel and browsed within the UI. This limit is configurable and was selected with input from our beta testers to provide a reasonable number of results that can be easily browsed without overwhelming an end user with dozens or hundreds of search result pages to sift through within the UI. The search result summary page contains the gene symbol, experimental platform, the probe identifier (for microarrays), experiment name, tissue or cell line type, species and fold change (degree of differential expression). In accordance with the remit of the NURSA website, the Molecules section of the site allows end users to browse and search NR, coregulator and ligands of interest. In addition, through Transcriptomine, an end user can also obtain molecule detail information about potential NR target genes by clicking the gene symbol link within a query results page. This action will open a Molecule Page, though unlike Molecule pages for NRs and coregulators, these pages do not contain NURSA-curated descriptions or seminal publications. The Tools section of the horizontal navigation pane underneath the NURSA banner contains a list of external tools that may be of interest to the NR research community–we welcome suggestions for links by e-mailing support@nursa.org.

#### Datasets

The All datasets page allows a user to filter, browse and search across all datasets from NDSPs. Recognizing the importance of a consistent, predictable browsing experience for the end user across the site, this page shares a design template and functionality with the All Molecules page. A browsable and sortable table contains information about NDSP-generated datasets, which again can be narrowed down using the filter module above. By clicking on the name of any dataset, end users can navigate to a dataset detail page in which a human curated description is provided along with information on the experimental platform type, tissue or cell lines used within the experiment, and species. Links to reagents created by NDSPs to generate the dataset and to relevant NR and coregulator Molecule Pages are present where applicable within the Reagent and Molecule sections.

#### Reagents

The layout and search, filter and browse functionalities of the Reagents page are similar to those described above. NDSPs have not only generated datasets available to the community, but also animal models, antibodies, cell lines, and primers. As before, reagents are summarized in a table that supports both browsing, where data in reagent Type, Molecule, reagent Name, Species or Source columns can be sorted by clicking on the column heading and filtering by specific reagent types, NR or coregulator molecules, or species. In addition, specific searches can be executed within the pane to the top right of the filter section. Reagent name hyperlinks within the table link to Reagent detail pages, whereas molecule symbol hyperlinks link to Molecule Pages. Each Reagent detail page contains summary information at top, such as genetic background for mouse animal models, or forward and reverse sequences for primers, and provides a link to Datasets with which they are associated.

#### Nuclear Receptor Signaling

One of the challenges facing the group during development of the original website was to advertise its existence to the global NR signaling community. Accordingly, we leveraged the popularity of PubMed to achieve this visibility by launching an Open Access electronic journal, *Nuclear Receptor Signaling*, developed according to the PubMed Central Extensible Markup Language (XML) Document Type Definition (DTD) [27]. By depositing full text XML files in PubMed Central we fulfilled the criteria for indexing in PubMed and, as such, exposed the NURSA website to user searching PubMed using NR-relevant terms. In addition to increasing visibility for the NURSA resource, *NRS* is a convenient, familiar medium for users to contribute primary research articles, reviews and perspectives to the NURSA knowledge mine. Articles containing global scale transcriptomic datasets will link to NURSA interfaces where gene lists associated with these datasets can be mined and interrogated in a convenient, intuitive manner (manuscript under preparation). *NRS* is indexed by Medline and has an internationally-recognized editorial board (www.nrsignaling.org).

#### Clinical

In response to survey input requesting inclusion of clinically-relevant data in NURSA 3.0, the Clinical section was added to meet this need. This section contains data from clinical trials, FDA-approved and experimental drugs, and diseases or phenotypes that have been linked to NRs, NR ligands or coregulators. Clinical trials from ClinicalTrials.gov are selected for inclusion based upon their utilization of NR ligands or NR-targeting drugs, or their purpose to target a condition or disease whose etiology has been linked to a NR or coregulator. Examples of clinical trials in NURSA 3.0 include those for: (i) breast, ovarian or prostate cancers that have been mapped to androgen receptor (AR), estrogen receptor α (ER α) or progesterone receptor (PR); (ii) treatment of chronic obstructive pulmonary diseases using the anti-inflammatory drug fluticasone, a glucocorticoid receptor (GR) agonist; and (iii) treatment of polycystic ovary syndrome (PCOS) that has been linked in OMIM, for example, to AR and ER. Because all trials within ClinicalTrials.gov are pulled down into the NURSA database, any clinical trial that is missed by our algorithm and subsequently reported by end users as being pertinent can be flagged for display once validated by the NURSA team. The Drugs subsection has similar look and feel to previously described sections, and allows end users to filter by targeted molecule gene symbol, disease condition or drug name, including synonyms recognized by PubChem and Drugbank. Drugs that are indexed include those for which a coregulator or NR has been annotated by Drugbank as being a carrier, enzyme, target or transporter. Records in this section contain expandable sections on Nomenclature for reviewing generic and brand name synonyms; Pharmacology to define indications, mechanisms of action, half life and other pharmacological characteristics; as well as the known Carriers, Enzymes, Targets and Transporters of a given drug. Where the drug is utilized within a NURSA-indexed clinical trial, information on the trial with a link to its page within the Hub is also provided.

#### Sponsored Research

To provide for the generation of novel ‘omics scale datasets by the NURSA Consortium, the NURSA Hub website publishes an annual solicitation for dedicated NURSA Data Source Projects (NDSPs). These research projects are expected to be innovative and to generate high dimensional datasets that will be shared by the Hub website. Solicitations are open to the entire community and undergo peer review by an external panel appointed by NIH institutional staff, and that does not include any member of the NURSA Consortium. Information on these awards is accessible via a prominent Funding link on the home page. The Funding section landing page provides an overview for this program with general details on a given NDSP, including the period of performance, total possible award, eligibility requirements and other information. While direct costs can vary, there is an option to continue for a second year should appropriate milestones be met. Current NDSP opportunities and ongoing/previous NDSP projects are linked within the NDSP announcements section of the overview. These pages also can be accessed by mousing over the Funding link within the horizontal navigation pane just under the NURSA banner, as can a listing of other funding resources and publications that have resulted from NDSPs.

#### Newswire and About

The @NursaTweets Twitter account is used to broadcast new NDSP funding opportunities, release of NURSA-supported datasets, new features of the Hub website and other similar updates to its community. It also follows other accounts that provide NR-relevant news, NIDDK and other relevant funding opportunities, and policy discussion with followers and within a continuous feed on the NURSA 3.0 website. The Newswire section box on the Homepage displays the latest tweet. Clicking this box opens the entire feed within the Newswire page. The About section contains contextual information on NURSA provided by its principal investigators and NIDDK program and scientific officers. This section also contains a Frequently Asked Questions guide to help end users understand the purpose of the site and how to use its data and tools. A highly popular feature that has been retained in the new version of the website is the animated tutorial “Nuclear Receptor Signaling: Concepts and Models” [28], which provides students and scientists unfamiliar with the field with in introduction to the history of NR signaling research.

### Digital Object Identifiers

The digital object identifier (DOI) system was developed to associate digital objects with an enduring, unique identifier that would persist irrespective of the location of the object. The system was developed by the International DOI Foundation, which co-ordinates the efforts of a number of DOI registration agencies, the first of which was CrossRef. The IDF also maintains a database mapping registered DOIs to their corresponding uniform resource locator, which greatly facilitates the maintenance of links between web resources and minimizes the possibility of broken connections. Reflecting its long-standing progressive commitment to digital publishing. NURSA was in 2004 the first NIH-funded biomedical data repository to adopt the DOI standard, and to begin depositing dataset metadata with CrossRef. In addition to datasets, NURSA DOIs are applied to NURSA 3.0 Molecule Pages, recognizing the intellectual effort invested in their creation and maintenance, and reflecting their enduring status as digital scholarly works.

## Discussion

Over the past 10–15 years, the field of NR signaling has generated a growing volume of global datasets that collectively describe sequences of NR and coregulator genes (genomics); the regulation by NRs, their ligands and coregulators of gene networks in specific target tissues (transcriptomics); protein-protein interactions required for the efficient function of NRs and coregulators (proteomics); specific sites of action of NRs in target gene promoters (cistromics); covalent modification of chromatin (epigenomics); and, more recently, specific functional endpoints in the form of regulation of cellular metabolic pathways (metabolomics). While traditional avenues of scientific publication must be maintained, there has been an increasingly recognized need for these to be complemented by web resources that can extract maximum collective leverage from these datasets by aggregating and annotating them in a quality-controlled fashion using standard terminologies and ontologies, then making them available to the NR research community for meta-analyses and *in silico* hypothesis generation. In response to this need, the Nuclear Receptor Signaling Atlas (NURSA) was initially funded in 2002, and has since matured into a comprehensive, stable and enduring website resource for researchers in this field and the wider scientific community.

### Impact of the NURSA database

Google PageRank is a proprietary scale used by Google to rank webpages that has been suggested to hold great promise for quantifying the impact of scientific publications with the advent of the Internet [29]. It uses the link structure of the Internet as an indicator of the value of an individual page, in essence interpreting a link from page A to page B as a vote by page A for page B. Critically, it takes into account not only the raw volume of votes (inbound links), but also analyzes the page that casts the vote. Inbound links from pages that are themselves "important" have more weight and help to make the target page "important". It has been suggested that PageRank should be more widely used as a measure of a journal’s prestige in addition to, or in place of, the ISI impact factor [30]. Table 2 shows the PageRank of a number of Internet biomedical research resources in comparison to that of NURSA. As can be seen, NURSA compares very favorably with a number of highly prominent and widely used resources, including ENCODE, Oncomine.org and Phosphosite, reflecting the quality of its annotation and programming, and the currency of its curation, compared to these resources.

**Table 2:** Google Page Rank of selected biomedical research resources.

Resource	Google Page Rank
UniProt	7
PharmGKB	7
PDB	7
Allen Brain Atlas	6
BioGPS	6
ENCODE	6
GPCRDB	6
**NURSA**	**6**
Molecular Libraries Program	6
Oncomine.org	6
Phosphosite	6
AR Gene Mutation DB	5
CTDBASE	5
NucleaRDB	5
IUPHARDB	5
HPRD	4
Nuclear Receptor Resource	4
Nuclear Receptor Cistrome	3

### Curational challenges

Curation represents a challenge for biological databases in any field, but particularly in highly multidisciplinary fields such as nuclear receptor signaling, which encompasses elements of biochemistry, cell biology, organic chemistry, physiology, pharmacology, structural biology, medical subspecialties and informatics. Given the realities of funding constraints, we have had to make strategic choices regarding where to focus our *de novo* curational activities, and where to rely on external databases for coverage in other areas. To ensure as far as possible that the data and information within NURSA are current and conformant to standards, we undertake a biannual aggregation of data from dozens of external public databases, with priority given to databases containing human curated content. Gaps in coverage arising from lags in curation in these databases are inevitable: it may be the case, for example, a specific molecular interaction has not yet been curated, or certain diseases have not yet been linked to a specific NR or coregulator, even if this information is already published in a paper. On other occasions, an external database may not employ the exact terminologies or synonyms that are indexed by NURSA. In these instances, a user of the NURSA site may not find what they are looking for. We strive to minimize the frequency with which this happens, and will continue to expand NURSA’s data coverage by adding new data sources, terminologies and synonyms from trusted sources.

### Involving the community

We greatly encourage the assistance of members of the community in assisting us in our curational efforts, and users can contact support@nursa.org to make suggestions or enquire about something that they are unable to locate on the site. In addition to the activities of the software-beta testing group, we have found that a successful solution to engaging the community has been the solicitation of manuscripts for NURSA’s peer reviewed journal, *Nuclear Receptor Signaling*. The format is familiar and flexible, it accommodates a broad spectrum of content and, most importantly, authors receive tangible credit for their contribution in the form of a MEDLINE-indexed publication. Although conceptually straightforward, the work involved in developing and maintaining wiki-based interfaces to allow members of the research community to contribute to maintaining the content of the Molecule Pages is prohibitive. Given the lack of academic incentive for such efforts, there is no guarantee of consistent quality of such contributions of the community, and any resulting errors or deficits would reflect poorly on the resource as a whole. Indeed, the initial promise of similar efforts in other resources [31] has unfortunately not been sustained in recent years. Recent initiatives that reposition NIH with respect to the curation and management of biomedical big data [14] indicate its intent to deal with this issue in a more systematic manner. We hope that the culture of biomedical research will evolve to the point where the curation of databases, by both full time curators and members of the academic community, is recognized less as philanthropy and more as serious scholarly work, to be rewarded with commensurate levels of formal academic credit.

### Future Directions

The overall goals of NURSA over the remainder of the current funding cycle are, firstly, to maintain and expand its data incorporation and annotation pipeline to increase the volume of data points in its NR signaling pathway database; and secondly, to incorporate, or develop *de novo*, data mining and visualization tools that will provide for detailed analysis by end users. Toward the first goal, we are planning a series of point releases to incorporate datasets and link to external sources, including NCBI-curated protein-protein interactions, reagent and animal model resources such AddGene and MGI, clinical cohort genomics data (ClinVar), additional protein data from BioGRID, and data from Malacards. Toward the second goal, a major effort for the next release of the NURSA Hub will be to update Transcriptomine to have the same look and feel as the existing NURSA 3.0 UI and to incorporate usability enhancements such as direct links from array probe or gene identifiers to the UCSC Genome Browser. In addition, we will leverage Cytoscape JS and D3.JS data visualization libraries to render interactive graphics such as summary statistics of the data within Transcriptomine, scatter charts of gene expression query results, expression data overlaid onto cellular pathways and other interactive solutions for visualizing Transcriptomine data points. Moreover, many of our end users have requested a feature in this mining tool to view relative expression levels in the context of transcription factor binding. Cistromic data pertinent to the NR field have been compiled within a beta version of the Nuclear Receptor Cistrome Data Browser (www.cistrome.org), and we will incorporate information from this resource and links to popular genome browsers cistromic and epigenomic tracks will be added in future releases. The value of NURSA as a domain-specific knowledgebase will be amplified by its inclusion in broader, disease-relevant, knowledgebases, specifically the NIDDK Information Network (www.dknet.org), creating opportunities for information flow into and out of the NR community.

Databases such as PubChem, CheBI and DrugBank are well known resources documenting the physical and chemical properties of small molecules. A significant gap in coverage of these resources, however, is the absence of data points documenting their regulation of gene expression in their target tissues. To address this deficit, we plan to develop collaborations with the curators of these resources to embed links in their NR ligand pages that will link directly to lists of the most highly induced and repressed genes in specific tissues and cell lines. In a second major collaborative effort, we are engaging scientific publishers and companies in the biomedical citation management spaces. The NIH Big Data To Knowledge (BD2K) initiative [14] is aimed at increasing the discoverability, re-usability and citability of NIH-funded biomedical datasets. Using BD2K funding, we are designing new data mining interfaces in Transcriptomine that will allow users to browse gene lists within a transcriptomic dataset and to discover related datasets. In addition, we are collaborating with publishers to embed links to these interfaces from relevant articles, thereby affording readers of articles to mine these datasets routinely and conveniently. Finally, to increase the citability of these datasets, we have collaborated with Thomson Reuters, the makers of the popular Endnote reference management tool, to allow users of Transcriptomine to easily incorporate dataset citations into manuscripts and research proposals.

In addition to these initiatives, a significant amount of our recent effort has been devoted to the development of application programming interfaces (APIs) which enable NURSA to share its *de novo* curated content with external databases in a scalable and sustainable manner. In the near future, these APIs will expose NURSA’s unique transcriptomic data points to dkNET, the aggregator search engine for NIDDK-funded research resources, as well as the Pharmacogenomic Knowledge Base (PharmGKB), which curates information on interactions between human genes and prescription drugs, many of which are NR agonists or antagonists. In return, we will pull in data from PharmGKB that will document phamacologically relevant polymorphisms in NR- and coregulator-encoding human genes. Finally, we will set up reciprocal data exchanges with the NIH Library of Integrated Network-based Cellular Signaling database (LINCS), which has developed network visualization tools, such as Enrichr, that can be used to interact with transcriptomic data points from NURSA and other sources. As ever, we welcome input from the community on other external data resources they would like to see integrated into the NURSA knowledge base.

## Conclusions

The advent of global discovery-driven ‘omics datasets–transcriptomic, proteomic, epigenomic and metabolomic—has placed at the disposal of researchers more opportunities than ever before to rapidly develop novel hypotheses, to validate experimental observations, or to make connections between different signaling pathways that were not previously apparent. The volume of these datasets has reached a point, however, where their effective management and distribution is arguably as important as the generation of novel datasets. The mission of the NURSA web resource is to ensure that datasets within the NR signaling field are archived, annotated, and exposed for data mining for the benefit of the entire cell signaling community, such that the considerable financial and scientific investment in these datasets can be fully realized. We invite input from the entire community on an ongoing basis so that our resource can be best positioned to assist researchers in managing the growing universe of ‘omics data points in this field.
